# Fucoidan/UVC Combined Treatment Exerts Preferential Antiproliferation in Oral Cancer Cells but Not Normal Cells

**DOI:** 10.3390/antiox11091797

**Published:** 2022-09-12

**Authors:** Ya-Ting Chuang, Jun-Ping Shiau, Ching-Yu Yen, Ming-Feng Hou, Jiiang-Huei Jeng, Jen-Yang Tang, Hsueh-Wei Chang

**Affiliations:** 1 Graduate Institute of Medicine, College of Medicine, Kaohsiung Medical University, Kaohsiung 80708, Taiwan; 2 Division of Breast Oncology and Surgery, Department of Surgery, Kaohsiung Medical University Hospital, Kaohsiung Medical University, Kaohsiung 80708, Taiwan; 3 School of Dentistry, Taipei Medical University, Taipei 11031, Taiwan; 4 Department of Oral and Maxillofacial Surgery, Chi-Mei Medical Center, Tainan 71004, Taiwan; 5 Department of Biomedical Science and Environmental Biology, College of Life Sciences, Kaohsiung Medical University, Kaohsiung 80708, Taiwan; 6 School of Dentistry, College of Dental Medicine, Kaohsiung Medical University, Kaohsiung 80708, Taiwan; 7 Department of Dentistry, Kaohsiung Medical University Hospital, Kaohsiung 80708, Taiwan; 8 Department of Dentistry, National Taiwan University Hospital, Taipei 100225, Taiwan; 9 School of Post-Baccalaureate Medicine, Kaohsiung Medical University, Kaohsiung 80708, Taiwan; 10 Department of Radiation Oncology, Kaohsiung Medical University Hospital, Kaoshiung Medical University, Kaohsiung 80708, Taiwan; 11 Center for Cancer Research, Kaohsiung Medical University, Kaohsiung 80708, Taiwan

**Keywords:** fucoidan, ultraviolet C, oral cancer, combined treatment, oxidative stress

## Abstract

Combined treatment is a promising anticancer strategy for improving antiproliferation compared with a single treatment but is limited by adverse side effects on normal cells. Fucoidan (FN), a brown-algae-derived polysaccharide safe food ingredient, exhibits preferential function for antiproliferation to oral cancer but not normal cells. Utilizing the preferential antiproliferation, the impacts of FN in regulating ultraviolet C (UVC) irradiation were assessed in oral cancer cells. A combined treatment (UVC/FN) reduced cell viability of oral cancer cells (Ca9-22 and CAL 27) more than single treatments (FN or UVC), i.e., 53.7%/54.6% vs. 71.2%/91.6%, and 89.2%/79.4%, respectively, while the cell viability of UVC/FN treating on non-malignant oral (S–G) was higher than oral cancer cells, ranging from 106.0 to 108.5%. Mechanistically, UVC/FN preferentially generated higher subG1 accumulation and apoptosis-related inductions (annexin V, caspases 3, 8, and 9) in oral cancer cells than single treatments. UVC/FN preferentially generated higher oxidative stress than single treatments, as evidenced by flow cytometry-detecting reactive oxygen species, mitochondrial superoxide, and glutathione. Moreover, UVC/FN preferentially caused more DNA damage (γH2AX and 8-hydroxy-2’-deoxyguanosine) in oral cancer cells than in single treatments. *N*-acetylcysteine pretreatment validated the oxidative stress effects in these UVC/FN-induced changes. Taken together, FN effectively enhances UVC-triggered antiproliferation to oral cancer cells. UVC/FN provides a promising potential for preferential and synergistic antiproliferation in antioral cancer therapy.

## 1. Introduction

Oral cancer is a typical head and neck malignancy with high morbidity and mortality [1,2,3] for both genders [4]. In addition to surgery, chemical and radiation therapies are applied to oral cancer patients in a single or combined treatment manner. However, these chemo- or radiotherapies are frequently associated with severe adverse effects [5].

Combined treatment with chemical drugs and natural products was utilized to improve antiproliferation effects of radiation against cancer cells [6,7,8]. Taking the example of ascorbate, it improves the ionizing radiation-induced DNA damage against glioblastoma cells [6]. Afatinib and berberine can synergistically promote radiosensitivity to lung [7] and liver cancer cells [8].

Alternatively, ultraviolet C (UVC), non-ionizing radiation also shows anticancer potential for combined treatment applications such as oral [9], colon [10], breast [11], and bladder [12] cancer cells. Cisplatin, for example, synergistically improved the antiproliferation effects of UVC on colon cancer cells [10]. However, cisplatin occasionally causes side effects of nephrotoxicity in clinical treatments [13]. Accordingly, the choice of anticancer drugs for enhancing UVC-inducing antiproliferation of cancer cells needs to consider the benefits of medications with low side effects.

Marine algae provide non-cytotoxic dietary food supplements [14]. Fucoidan (FN), a fucose-rich polysaccharide isolated from several brown algae, is a safe food ingredient, classified as GRAS by the United States Food and Drug Administration (FDA) [15]. FN exhibits diverse functions for inhibiting inflammation [16], bacterial growth [17], and cancer proliferation [18,19]. The antiproliferation effects of FN have been reported for oral [20], breast [21], and bladder [22] cancer cells. Notably, FN shows preferential killing to oral cancer cells but not to non-malignant cells. Accordingly, there are no side effects of FN to be expected.

Recently, the combined treatment of anticancer agents with FN was reported using cisplatin [23], gefitinib [24], and vitamin C [25] for lung, breast, and colon cancer cells, respectively, while the combined treatment of anticancer therapy with FN in oral cancer was rarely applied, particularly together with UVC irradiation.

This study assesses the antiproliferation effects and mechanisms of UVC/FN in oral cancer cells. To clarify the detailed mechanism, both oral cancer and non-malignant oral cells were chosen to assess the status of oxidative stress, apoptosis, and DNA damage of UVC/FN treatment.

## 2. Materials and Methods 

### 2.1. Reagents and UVC Irradiation

*Fucus vesiculosus*-derived FN was acquired from Carbosynth (Compton, Berkshire, UK). Oxidative stress scavenger, 10 mM *N*-acetylcysteine (NAC) [26,27,28] (Sigma-Aldrich; St. Louis, MO, USA), was pretreated for 1 h and co-treated with FN for 24 h. Both FN and NAC were prepared in 1 × PBS solution.

After medium aspiration, cells were irradiated with a germicidal UVC lamp (254 nm) (10 or 15 J/m^2^) for 10 or 15 s at a rate of 1 J/m^2^/s in a laminar flow hood. This energy rate was detected by a UV radiometer (UVP, San Gabriel, CA, USA) before UVC irradiation [9]. Control cells followed the same protocol without UVC irradiation. After UVC irradiation, cells were treated with FN.

### 2.2. Cell Culture and MTS Viability

Oral cancer (Ca9-22 and CAL 27) cell lines were acquired from the HSRRB Cell Bank (Osaka, Japan) and ATCC (Manassas, VA, USA). A non-malignant gingival epithelial Smulow–Glickman (S–G) cell line [29,30], generally applied for assessing the drug safety of oral cells [31], was included. They were maintained in DMEM/F-12 (3:2) with P/S antibiotics and 10% fetal bovine serum (Gibco; Grand Island, NY, USA). Cell viability at 24 h was determined by Promega’s MTS assay (Madison, WI, USA) and read by a multiplate reader at 490 nm [20].

The synergy (α) of a combined UVC/FN treatment was determined as previously described [32], i.e., α = the viability fraction (UVC) × the viability fraction (FN)/the viability fraction (UVC/FN). The relationship of additive, synergistic, or antagonistic antiproliferation was α = 1, > 1 and < 1, respectively.

### 2.3. Cell Cycle

Cellular DNA was stained with 7-aminoactinomycin D (7AAD, 1 μg/mL) (Biotium Inc., Hayward, CA, USA) and incubated at 37 °C for 30 min. The intensity of DNA levels was inspected by a Guava easyCyte flow cytometer (Luminex, TX, USA), and data were processed by Flow Jo 10 software (Becton-Dickinson, Franklin Lakes, NJ, USA) [20].

### 2.4. Apoptosis

Annexin V/7AAD [33] analytical method was used to monitor apoptosis by the commercial kit (Strong Biotech Corp, Taipei, Taiwan). The intensities of annexin V/7AAD were measured by Guava easyCyte flow cytometer.

Caspase (Cas) 3, Cas 8, and Cas 9 flow cytometry analyses were designed to detect their activation degrees for the executor, extrinsic, and intrinsic caspases [34]. Peptide-based kits (OncoImmunin; Gaithersburg, MD, USA) were conducted to measure Cas 3, Cas 8, and Cas 9 activities by flow cytometry [35]. A 10 μM peptide solution was diluted in 1:1000 for incubation at 37 °C for 1 h. Activated Cas 3, Cas 8, and Cas 9 can digest their specific substrates (PhiPhiLux-G1D2, CaspaLux8-L1D2, and CaspaLux9-M1D2). Subsequently, the digested substrates could generate fluorescence and be analyzed by flow cytometry.

### 2.5. Oxidative Stress

Reactive oxygen species (ROS) [36] and mitochondrial superoxide (MitoSOX) [20] were chosen to detect the status of oxidative stress after drug treatment. A total of 100 nM 2′,7′-dichlorodihydrofluorescein diacetate (H_2_DCF-DA) (Sigma-Aldrich) and 50 nM MitoSOX™ Red (Thermo Fisher Scientific, Carlsbad, CA, USA) were used to detect ROS and MitoSOX at 37 °C for 30 min, respectively. The intensities of these oxidative stresses were measured by a Guava easyCyte flow cytometer.

### 2.6. Glutathione (GSH)

GSH was chosen to detect the status of cellular antioxidant levels after drug treatment. 5-chloromethylfluorescein diacetate (CMF-DA) (Thermo Fisher Scientific, Carlsbad, CA, USA) (5 μM, 20 min) [20] was used at 37 °C for 30 min to detect GSH levels. The intensities of GSH were measured by a Guava easyCyte flow cytometer.

### 2.7. DNA Damages

Cell fixation was required before antibody reaction in detecting DNA damage. Monoclonal antibodies for mouse p-Histone H2A.X (Ser 139) (Santa Cruz Biotechnology, Santa Cruz, CA, USA) (1:500) [37] and Alexa 488-secondary antibody were chosen for the detection of DNA double-strand breaks in the presence of 5 μg/mL 7AAD. Finally, the γH2AX and 7AAD intensities were monitored by flow cytometry. To investigate oxidative DNA damage, the monoclonal antibody against mouse 8-hydroxy-2′-deoxyguanosine (8-OHdG)-FITC (Santa Cruz Biotechnology) (1:10,000) [20] was incubated with the fixed cells. Finally, the 8-OHdG-FITC intensities were inspected by flow cytometry.

### 2.8. Statistics

In multiple comparisons, the significance of the difference was determined by ANOVA analysis combined with the Tukey HSD Post Hoc Test (JMP 14 software, SAS Institute Inc., Cary, NC, USA) [20]. Data were shown as means ± SD (*n* = 3). Lower-case letters were assigned by JMP software to determine the significance of results where data with non-overlapping letters represent a significant result.

## 3. Results

### 3.1. UVC/FN versus Single Treatment on Antiproliferation

**Figure 1:**
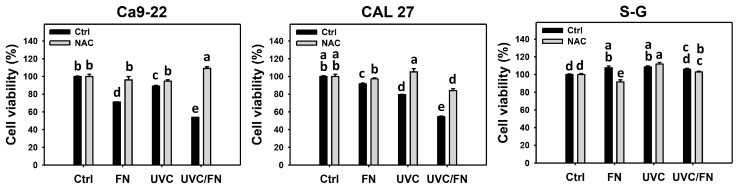
Cell viability determination. There are four kinds of treatment for oral cancer (Ca9-22 and CAL 27) and non-malignant oral (S–G) cells: control, FN (300 μg/mL), UVC (10 J/m^2^ for Ca9-22; 15 J/m^2^ for CAL 27 and S–G), and UVC/FN (10 J/m^2^/300 μg/mL for Ca9-22; 15 J/m^2^/300 μg/mL for CAL 27 and S–G). NAC indicates that cells were pretreated with 10 mM NAC. Their viabilities were determined by MTS assay after 24 h treatment. Data are provided as means ± SD (*n* = 3). The low-case letters were assigned by JMP software to determine significant differences. Significant differences were indicated by non-overlapping, low-case letters (*p* < 0.05). For the example of Ca9-22 cells, the low-case letters for control, FN, UVC, and UVC/FN, showing “b, d, c, and e” significantly differ from each other because the letters are not overlapping. Moreover, UVC/FN for control and NAC showing “e and a” indicates significant differences. In contrast, the controls for non-treatment and NAC only showing the same letter “b” indicate non-significant results.

Antiproliferation effects were compared between combined and single treatment (UVC and/or FN) by 24 h MTS assays. In oral cancer cells (Ca9-22), UVC/FN demonstrated lower viability than a single treatment (300 μg/mL FN or 10 J/m^2^ UVC), i.e., 53.7% vs. 71.2% and 89.2%, respectively (Figure 1). In oral cancer cells (CAL 27), UVC/FN demonstrated lower viability than a single treatment (300 μg/mL FN or 15 J/m^2^ UVC), i.e., 54.6% vs. 91.6% and 79.4%, respectively. In non-malignant oral cells (S–G), UVC/FN demonstrated similar viability to a single treatment (300 μg/mL FN or 15 J/m^2^ UVC), i.e., 106.0% vs. 107.6% and 108.5%, respectively. The synergy (α-values) of UVC/FN in Ca9-22 and CAL 27 cells were 1.20 and 1.33 (Figure 1), indicating that UVC/FN exhibited synergistic antiproliferation on oral cancer cells. Moreover, the viability of S–G cells was higher than that of oral cancer cells in the UVC/FN treatment. These results indicate that UVC/FN preferentially inhibits the proliferation of oral cancer cells but shows little change on non-malignant cells.

Utilizing *N*-acetylcysteine (NAC) pretreatment, the impact of oxidative stress in synergistic antiproliferation effects of UVC/FN was assessed. NAC effectively increased the viabilities of UVC and/or FN treatments acting on oral cancer and non-malignant cells (Figure 1). Therefore, UVC/FN demonstrates the synergistic antiproliferation of oral cancer cells relying on oxidative stress.

### 3.2. UVC/FN versus Single Treatment on SubG1 Increment

**Figure 2:**
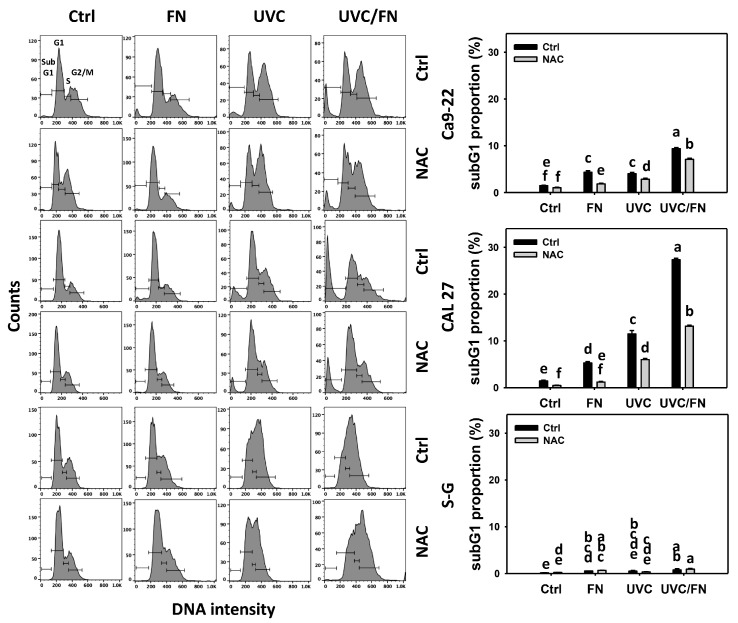
Cell cycle determination. There are four kinds of treatments for oral cancer (Ca9-22 and CAL 27) and non-malignant oral (S–G) cells: control, FN (300 μg/mL), UVC (10 J/m^2^ for Ca9-22; 15 J/m^2^ for CAL 27 and S–G), and UVC/FN (10 J/m^2^/300 μg/mL for Ca9-22; 15 J/m^2^/300 μg/mL for CAL 27 and S–G). NAC indicates that cells were pretreated with 10 mM NAC. Their cell cycle changes were evaluated by flow cytometry after a 24 h treatment. Data are indicated as means ± SD (*n* = 3). The low-case letters were assigned by JMP software to determine their significance. It significantly differs when the low-case letters are not overlapping (*p* < 0.05).

The cell-cycle-modulating effects were compared between combined and single treatment (UVC and/or FN) through 24 h 7AAD assays. In oral cancer cells (Ca9-22 and CAL 27), UVC/FN demonstrated higher subG1% than single treatments (FN or UVC) (Figure 2). In contrast, minor subG1% changes occurred in non-malignant cells (S–G) irrespective of whether in combined or single treatments. These results indicate that UVC/FN preferentially induces subG1 increment to oral cancer cells but shows little change on non-malignant cells.

Utilizing NAC pretreatment, the impact of oxidative stress on the synergistic subG1 increasing effects of UVC/FN was assessed. NAC effectively decreased the subG1% of UVC and/or FN treatments in oral cancer cells (Figure 2). Therefore, UVC/FN demonstrates the synergistic subG1 increment of oral cancer cells relying on oxidative stress.

### 3.3. UVC/FN versus Single Treatment on Annexin V Increment

**Figure 3:**
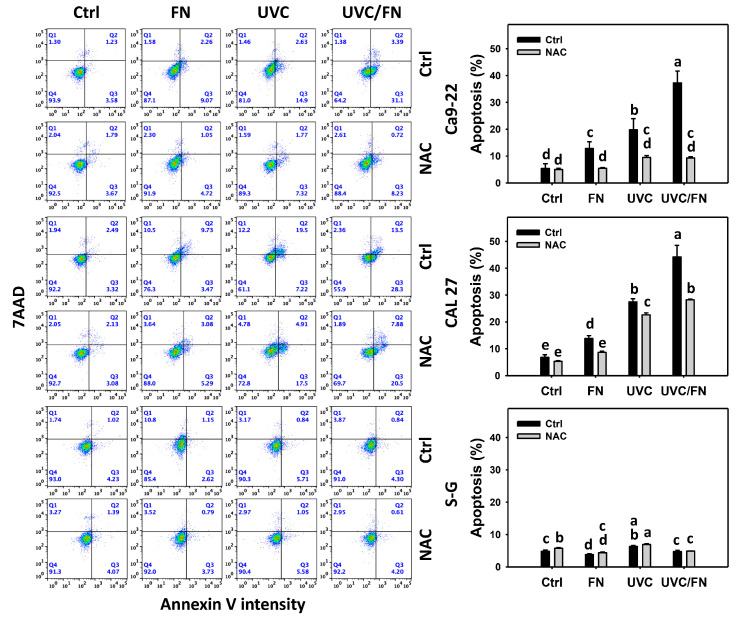
Annexin V/7AAD determination. There are four kinds of treatments for oral cancer (Ca9-22 and CAL 27) and non-malignant oral (S–G) cells: Control, FN (300 μg/mL), UVC (10 J/m^2^ for Ca9-22; 15 J/m^2^ for CAL 27 and S–G), and UVC/FN (10 J/m^2^/300 μg/mL for Ca9-22; 15 J/m^2^/300 μg/mL for CAL 27 and S–G). NAC indicates that cells were pretreated with 10 mM NAC. Their intensity changes were evaluated by flow cytometry after a 24 h treatment. Annexin V (+)/7AAD (−) and (+) populations were defined as apoptosis (+). Data indicated as means ± SD (*n* = 3). The low-case letters were assigned by JMP software to determine their significance. Significant differences are indicated by non-overlapping, lower-case letters (*p* < 0.05).

The apoptosis-modulating effects were compared between combined and single treatment (UVC and/or FN) by a 24 h annexin V/7AZD assays. In oral cancer cells, UVC/FN demonstrated higher annexin V (+)% than single treatment (FN or UVC) (Figure 3). In contrast, annexin V (+)% showed minor changes in non-malignant oral cells in combined or single treatments. These results indicate that UVC/FN preferentially induced apoptosis in oral cancer cells but showed little change on non-malignant cells.

Utilizing NAC pretreatment, the impact of oxidative stress in synergistic annexin V (+)% increasing effects of UVC/FN was assessed. NAC effectively decreased the annexin V (+)% of UVC and/or FN treatments acting on oral cancer cells (Figure 3). Therefore, UVC/FN demonstrates synergistic apoptosis of oral cancer cells relying on oxidative stress.

### 3.4. UVC/FN versus Single Treatment on Caspase 3 Activation

**Figure 4:**
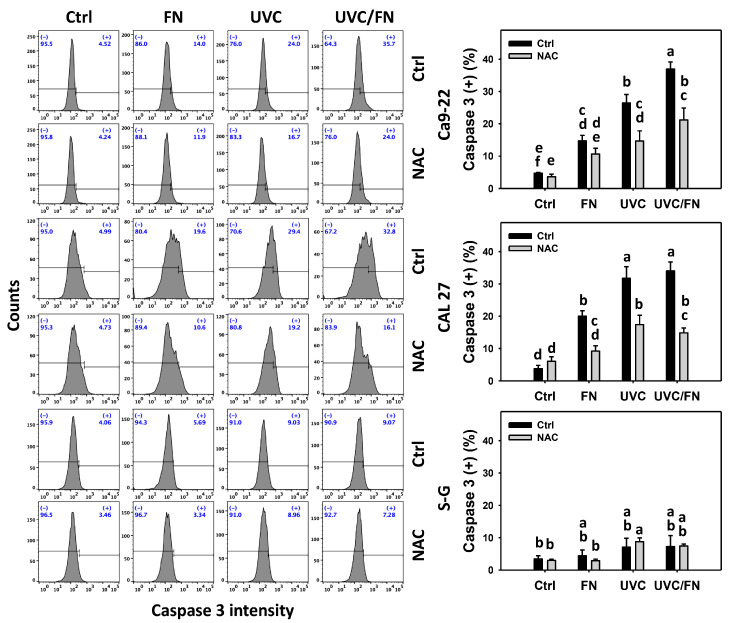
Caspase 3 activity determination. There are four kinds of treatments for oral cancer (Ca9-22 and CAL 27) and non-malignant oral (S–G) cells: control, FN (300 μg/mL), UVC (10 J/m^2^ for Ca9-22; 15 J/m^2^ for CAL 27 and S–G), and UVC/FN (10 J/m^2^/300 μg/mL for Ca9-22; 15 J/m^2^/300 μg/mL for CAL 27 and S–G). NAC indicates that cells were pretreated with 10 mM NAC. Their intensity changes were evaluated by flow cytometry after 24 h treatment. Caspase 3 (+) populations are indicated with (+). Data are provided as means ± SD (*n* = 3). Significant differences are indicated when lower-case letters are not overlapping (*p* < 0.05).

The apoptosis activation effects were compared between combined and single treatment (UVC and/or FN) with 24 h caspase 3 assays. In oral cancer cells, UVC/FN demonstrated higher caspase 3 (+)% than single treatment (FN or UVC) (Figure 4). In contrast, caspase 3 (+)% showed minor changes in non-malignant oral cells in combined or single treatments. The results indicate that UVC/FN preferentially induces apoptosis in oral cancer cells but shows little effect on non-malignant cells.

Utilizing a NAC pretreatment, the impact of oxidative stress in synergistic caspase 3 (+)% increasing effects of UVC/FN was assessed. NAC effectively decreased the caspase 3 (+)% of UVC and/or FN treatments acting on oral cancer cells (Figure 4). Therefore, UVC/FN demonstrate synergistic activation of apoptosis signaling of oral cancer cells relying on oxidative stress.

### 3.5. UVC/FN versus Single Treatment on Extrinsic and Intrinsic Caspase Activations

**Figure 5:**
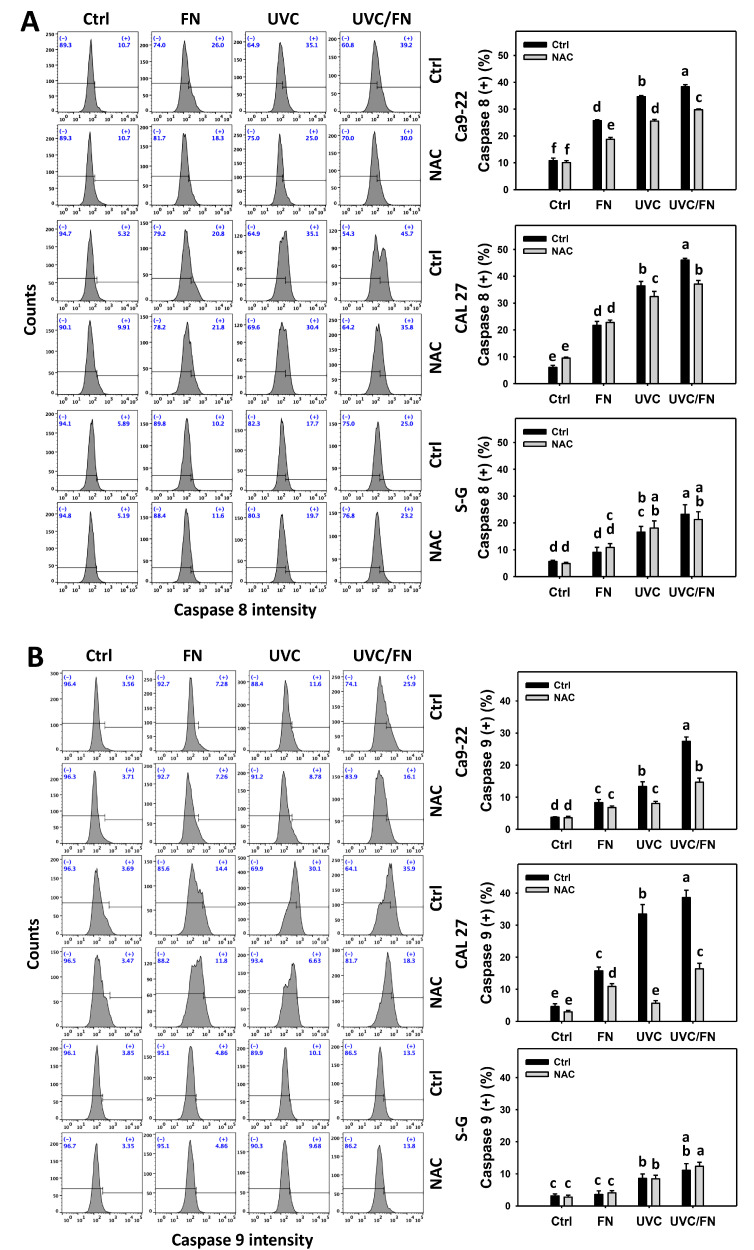
Extrinsic and intrinsic signaling determination. (**A**) Caspase 8. (**B**) Caspase 9. There are four kinds of treatments for oral cancer and non-malignant oral (S–G) cells: control, FN (300 μg/mL), UVC (10 J/m^2^ for Ca9-22; 15 J/m^2^ for CAL 27 and S–G), and UVC/FN (10 J/m^2^/300 μg/mL for Ca9-22; 15 J/m^2^/300 μg/mL for CAL 27 and S–G). NAC indicates that cells were pretreated with 10 mM NAC. Their intensity changes were evaluated by flow cytometry after 24 h treatment. Caspase 8 (+) and caspase 9 (+) populations are indicated by (+). Data are given as means ± SD (*n* = 3). Lower-case letters indicate significant differences when non-overlapping (*p* < 0.05).

The activation effects of extrinsic (caspase 8) and intrinsic (caspase 9) apoptosis signaling were compared between combined and single treatment (UVC and/or FN) at 24 h caspase 8/9 assays. In oral cancer cells, UVC/FN demonstrated higher caspases 8 and 9 (+)% than single treatment (FN or UVC) (Figure 5A,B). In contrast, caspases 8 and 9 (+)% showed minor changes in non-malignant oral cells in combined or single treatments. These results indicate that UVC/FN preferentially induces extrinsic and intrinsic apoptosis signaling to oral cancer cells but shows low change on non-malignant cells.

Utilizing NAC pretreatment, the impact of oxidative stress in synergistic caspases 8 and 9 (+)% increasing the effects of UVC/FN was assessed. NAC effectively decreased caspases 8 and 9 (+)% of UVC and/or FN treatments acting on oral cancer cells (Figure 5A,B). Therefore, UVC/FN demonstrates synergistic activation of extrinsic and intrinsic apoptosis signaling of oral cancer cells relying on oxidative stress.

### 3.6. UVC/FN versus Single Treatment on ROS/MitoSOX

**Figure 6:**
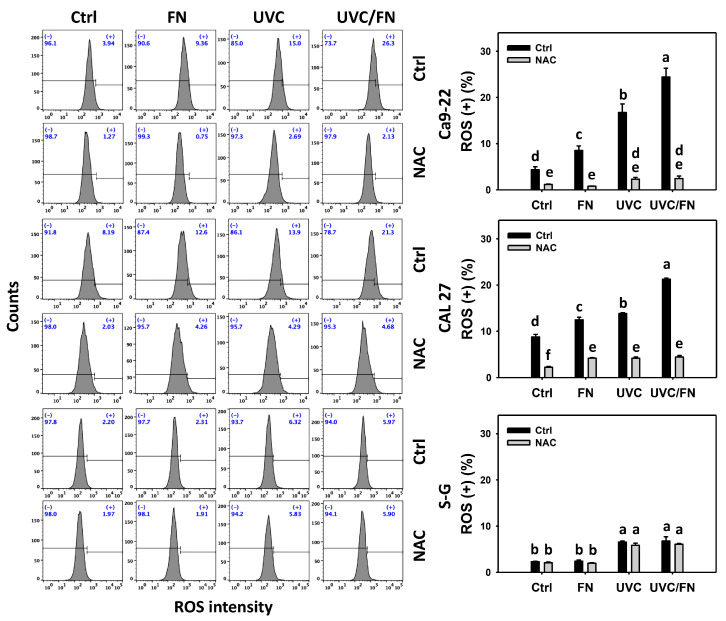
ROS determination. There are four kinds of treatments for oral cancer and non-malignant oral (S–G) cells: control, FN (300 μg/mL), UVC (10 J/m^2^ for Ca9-22; 15 J/m^2^ for CAL 27 and S–G), and UVC/FN (10 J/m^2^/300 μg/mL for Ca9-22; 15 J/m^2^/300 μg/mL for CAL 27 and S–G). NAC indicates that cells were pretreated with 10 mM NAC. Their intensity changes were evaluated by flow cytometry after 24 h treatment. ROS (+) populations are indicated with (+). Data are provided as means ± SD (*n* = 3). Significant differences are indicated by non-overlapping, lower-case letters (*p* < 0.05).

**Figure 7:**
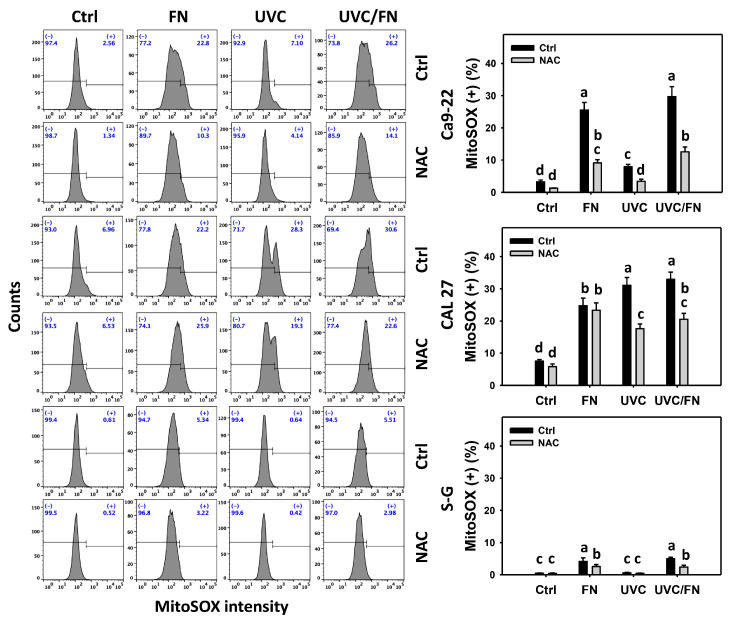
MitoSOX determination. There are four kinds of treatments for oral cancer and non-malignant oral (S–G) cells: control, FN (300 μg/mL), UVC (10 J/m^2^ for Ca9-22; 15 J/m^2^ for CAL 27 and S–G), and UVC/FN (10 J/m^2^/300 μg/mL for Ca9-22; 15 J/m^2^/300 μg/mL for CAL 27 and S–G). NAC indicates that cells were pretreated with 10 mM NAC. Their intensity changes were evaluated by flow cytometry after a 24 h treatment. MitoSOX (+) populations are indicated with (+). Data are given as means ± SD (*n* = 3). They significantly differ when the lower-case letters are not overlapping (*p* < 0.05).

Oxidative stress-modulating effects were compared between combined and single treatment (UVC and/or FN) at 24 h ROS and MitoSOX assays. In oral cancer cells, UVC/FN demonstrated higher ROS and MitoSOX (+)% than a single treatment (FN or UVC) (Figure 6 and Figure 7). In contrast, ROS and MitoSOX (+)% showed minor changes in non-malignant oral cells in combined or single treatments. These results indicate that UVC/FN preferentially induces oxidative stress on oral cancer cells but shows little change on non-malignant cells.

Utilizing NAC pretreatment, the impact of oxidative stress in synergistic ROS and MitoSOX (+)% increasing the effects of UVC/FN was assessed. NAC effectively decreased the ROS and MitoSOX (+)% of UVC and/or FN treatments acting on oral cancer cells (Figure 6 and Figure 7). Therefore, UVC/FN demonstrates the synergistic oxidative stress generation of oral cancer cells.

### 3.7. UVC/FN versus Single Treatment on GSH Depletion

**Figure 8:**
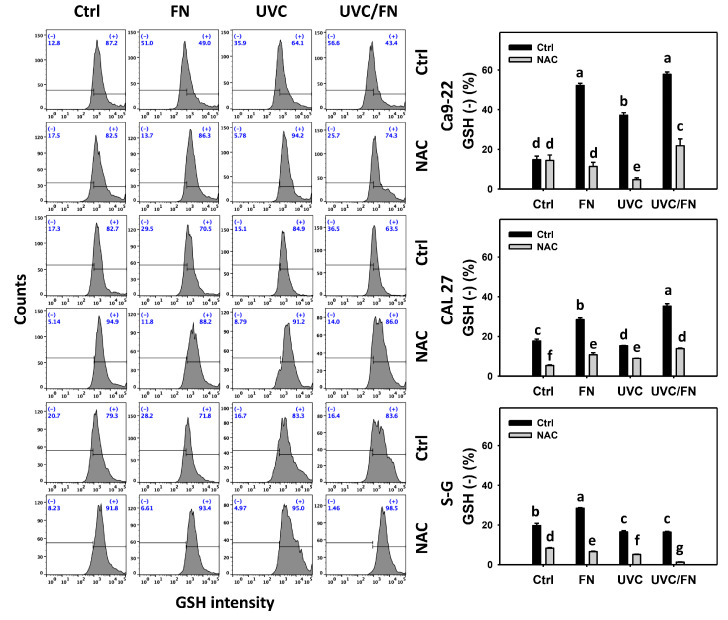
GSH determination. There are four kinds of treatmentss for oral cancer and non-malignant oral (S–G) cells: control, FN (300 μg/mL), UVC (10 J/m^2^ for Ca9-22; 15 J/m^2^ for CAL 27 and S–G), and UVC/FN (10 J/m^2^/300 μg/mL for Ca9-22; 15 J/m^2^/300 μg/mL for CAL 27 and S–G). NAC indicates that cells were pretreated with 10 mM NAC. Their intensity changes were evaluated by flow cytometry after 24 h treatment. GSH (−) populations are indicated with (−). Data are given as means ± SD (*n* = 3). Lower-case letters indicate significant differences when non-overlapping (*p* < 0.05).

The antioxidant modulating effects were compared between combined and single treatment (UVC and/or FN) at 24 h GSH assays. In oral cancer cells, UVC/FN demonstrated higher GSH (−)% than single treatments (FN or UVC) (Figure 8). In contrast, GSH (−)% showed minor changes in non-malignant oral cells in combined or single treatments. These results indicate that UVC/FN preferentially induces oxidative stress on oral cancer cells but shows little change on non-malignant cells.

Utilizing NAC pretreatment, the function of oxidative stress in synergistic GSH (−)% increasing the effects of UVC/FN was assessed. NAC effectively decreased the GSH (−)% of UVC and/or FN treatments acting on oral cancer cells (Figure 8). Therefore, UVC/FN demonstrates the synergistic oxidative stress generation of oral cancer cells.

### 3.8. UVC/FN versus Single Treatment on DNA Damage 

**Figure 9:**
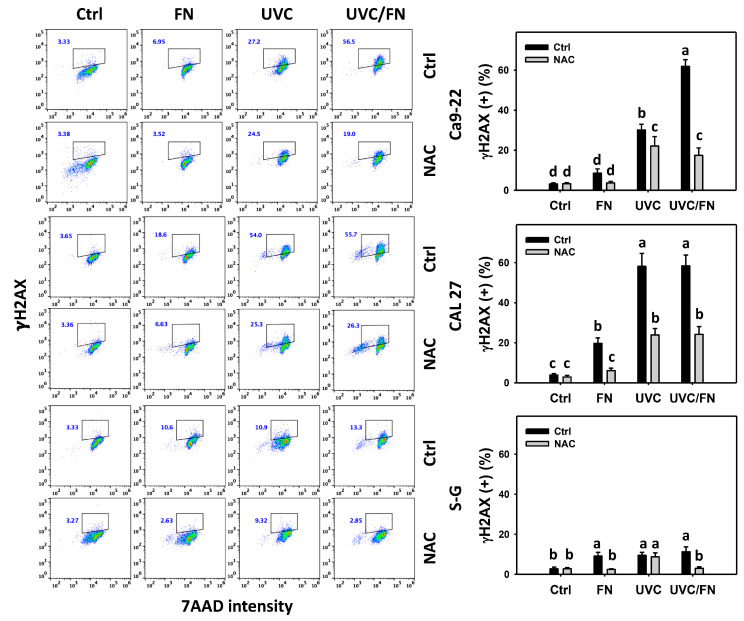
γH2AX determination. There are four kinds of treatments for oral cancer and non-malignant oral (S–G) cells: control, FN (300 μg/mL), UVC (10 J/m^2^ for Ca9-22; 15 J/m^2^ for CAL 27 and S–G), and UVC/FN (10 J/m^2^/300 μg/mL for Ca9-22; 15 J/m^2^/300 μg/mL for CAL 27 and S–G). NAC indicates that cells were pretreated with 10 mM NAC. Their intensity changes were evaluated by flow cytometry after a 24 h treatment. γH2AX (+) populations are indicated with (+). Data are provided as means ± SD (*n* = 3). The lower-case letters indicate significant differences when not overlapping (*p* < 0.05).

**Figure 10:**
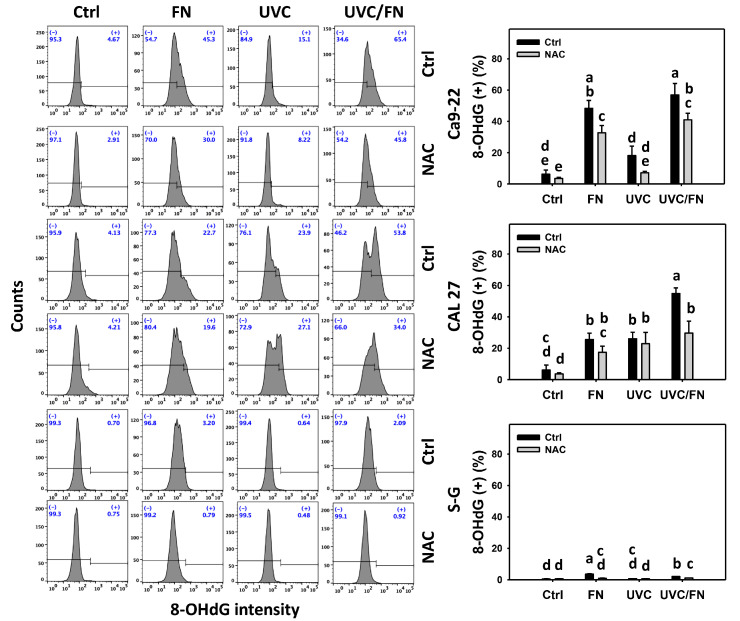
8-OHdG determination. There are four kinds of treatments for oral cancer (Ca9-22 and CAL 27) and non-malignant oral (S–G) cells: control, FN (300 μg/mL), UVC (10 J/m^2^ for Ca9-22 and S–G; 15 J/m^2^ for CAL 27), and UVC/FN (10 J/m^2^ for Ca9-22 and S–G; 15 J/m^2^ for CAL 27 and 300 μg/mL for all cell types). NAC indicates that cells were pretreated with 10 mM NAC. Their intensity changes were evaluated by flow cytometry after 24 h treatment. 8-OHdG (+) populations are indicated with (+). Data are given as means ± SD (*n* = 3). Lower-case letters determine their significance. Significant differences are indicated by non-overlapping, lower-case letters (*p* < 0.05).

The DNA damage-modulating effects were compared between combined and single treatments (UVC and/or FN) at 24 h γH2AX and 8-OHdG assays. In oral cancer cells, UVC/FN demonstrated higher γH2AX and 8-OHdG (+)% than single treatments (FN or UVC) (Figure 9 and Figure 10). In contrast, γH2AX and 8-OHdG (+)% showed more minor changes in non-malignant oral cells than in combined or single treatments. These results indicate that UVC/FN preferentially induces oxidative stress on oral cancer cells but shows little effect in non-malignant cells.

Utilizing NAC pretreatment, the impact of oxidative stress in synergistic γH2AX and 8-OHdG (+)% increasing the effects of UVC/FN was assessed. NAC effectively decreased the γH2AX and 8-OHdG (+)% of UVC and/or FN treatments acting on oral cancer cells (Figure 9 and Figure 10). Therefore, UVC/FN demonstrates synergistic effects by oxidative stress generation in oral cancer cells.

## 4. Discussion

The antiproliferation-enhancing ability of a combined FN/UVC treatment had not been reported before this study. Therefore, the present study explored several UVC/FN-associated mechanisms between oral cancer and non-malignant oral cells.

FN is known as an effective anticancer enhancer. It was applied to combined treatments with several anticancer drugs. Taking the example of breast cancer cells, FN combined with cisplatin, tamoxifen, paclitaxel [38], and doxorubicin [23] exhibit synergistic antiproliferation effects. Combined treatment of FN with tyrosine kinase inhibitor lapatinib suppresses more proliferation of esophageal cancer cells (OE33) [39]. FN also improves drug sensitivity to gefitinib, the epidermal growth factor receptor inhibitor acting on lung cancer cells [24]. 

However, drug-induced adverse effects of these clinical drugs were reported before. For example, the side effects of cisplatin [40], tamoxifen [41], paclitaxel [42], doxorubicin [43], lapatinib [44], and gefitinib [45] were reported. The drug safety of the above drugs treated in combination with other antiproliferation treatments was not investigated using non-malignant cells.

X-ray and UVC provide alternative radiation therapies to suppress the proliferation of several cancer types, such as oral [9], colon [10], breast [11], and bladder [12] cancer cells. UVC has been applied to combined treatment with several anticancer therapies [10,12,46]. For example, cisplatin enhances the UVC-induced antiproliferation of colon cancer cells [10]. However, this study did not consider the treatment safety of non-malignant cells. Recently, a preferential antiproliferation chemical CHW09 combined with UVC irradiation exhibited synergistic effects on inhibiting the proliferation of oral cancer cells but not on non-malignant cells [46]. A combined treatment of UVC/FN was performed in the present study. The design strategy is based on the combination of low-dose UVC and low-dose FN acting on oral cancer cells and compared all responses with non-malignant oral cells. In our study, UVC/FN showed no cytotoxicity to non-malignant cells but preferential antiproliferation to oral cancer cells (Figure 1).

Oxidative stress promotes the antiproliferation of cancer cells [47,48]. Combining different oxidative stress-modulating treatments may evoke synergistic oxidative stress, leading to synergistic antiproliferation [49,50,51]. UVC is an oxidative stress inducer [46,50,51,52,53] in cancer cells. Moreover, FN also functions as an ROS and MitoSOX inducer in oral cancer cells [20]. As expected, the oxidative stress (ROS and MitoSOX) was cooperatively induced by UVC/FN in oral cancer cells (Figure 6 and Figure 7). Notably, UVC/FN shows higher oxidative stress in oral cancer cells than in non-malignant ones. Hence, UVC/FN exhibits preferential and synergistic oxidative stress for oral cancer cells but not non-malignant cells.

Antioxidants and prooxidants govern redox homeostasis. When antioxidants are downregulated, prooxidants increase to higher levels than antioxidants, generating cellular oxidative stress. For example, emodin elevated ROS and decreased GSH in gallbladder cancer cells [54]. Alantolactone induced ROS and apoptosis by GSH depletion in glioblastoma cells [55]. FN also elicited ROS generation in oral cancer accompanied by GSH depletion [20]. UV irradiations such as UVB [56] and UVC [57] induced GSH depletion and the generation of ROS. Accordingly, FN/UVC in oral cancer cells caused more GSH depletion than a single treatment (UVC or FN), as evidenced by this present study (Figure 8). In contrast, non-malignant oral cells showed lower GSH depletion more than oral cancer cells. Therefore, UVC/FN exhibits a preferential and synergistic GSH depletion in oral cancer cells but not in non-malignant cells. This also contributes to the preferential oxidative stress of UVC/FN during oral cancer treatment.

In addition to antiproliferation, oxidative stress triggers apoptosis [58,59] and DNA damage [60,61]. UVC [52,53,62,63] and FN [20] represent the apoptosis inducers in cancer cells. Moreover, UVC [46] and FN [20] also cause DNA damage, as indicated by γH2AX and 8-OHdG assays. Consistently, this character of synergistic oxidative stress evoked by UVC/FN causes several oxidative stress-dependent mechanisms such as extrinsic and intrinsic apoptosis (Figure 5) as well as DNA double-strand breaks and oxidative DNA damage, i.e., γH2AX and 8-OHdG (Figure 9 and Figure 10). Additionally, the caspases 3, 8, and 9 activations are higher in oral cancer cells than in non-malignant cells. However, two oral cancer cell lines showed slightly different responses to UVC/FN acting on caspases 8 and 9 activations. In UVC/FN, CAL 27 cells showed higher annexin V, caspase 8, and 9 activations than Ca9-22 cells (Figure 3 and Figure 5), particularly for caspase 9, although their cell viabilities were similar (Figure 1). The differential responses to UVC/FN may be derived from their optimal treatment conditions being different, i.e., UVC/FN (10 J/m^2^/300 μg/mL for Ca9-22; 15 J/m^2^/300 μg/mL for CAL 27). In this evidence, it is possible that the apoptosis-inducible effects only partly contribute to the synergistic antiproliferation of UVC/FN in oral cancer treatments.

Since NAC is a GSH precursor [64], NAC pretreatment may replenish the GSH pool to drug-induced GSH depletion. Consistently, UVC and/or FN-induced GSH depletion in oral cancer cells was recovered by NAC (Figure 8). Furthermore, the dependence of oxidative stress in UVC/FN-induced antiproliferation and mechanism was validated by a NAC pretreatment. The NAC pretreatment of oral cancer cells recovered the UVC/FN-induced synergistic antiproliferation, oxidative stress, apoptosis, and DNA damage.

UVC exhibits low-penetrating but influential sterilization and DNA damage [65]. Hence, UVC irradiation is limited to surface tumors such as squamous cell carcinoma, accounting for 90% of oral cancer cells [66]. UVC also suppresses tumor growth in animal studies [67] but needs to test the functional depth layers by diacetylene-based film dosimeters as UVB phototherapy [67].

## 5. Conclusions

FN generates greater antiproliferation, oxidative stress, and DNA damage to oral cancer cells than non-malignant cells [20]. UVC also generates oxidative stress [46,50,51], apoptosis [62], and DNA damage [46]. However, a combined treatment of UVC/FN had not been investigated for anticancer, particularly for antioral cancer cells. The present study shows a more promising effect of a combined UVC/FN treatment to inhibiting oral cancer cells than a single treatment (UVC or FN). The results validate that UVC/FN exhibited synergistic functions for generating more antiproliferation, oxidative stress, GSH depletion, apoptosis, and DNA damage in oral cancer cells than in non-malignant cells involving oxidative stress-modulating mechanisms. Therefore, UVC/FN offers the potential for a combined treatment for antioral cancer cells showing no adverse effects on non-malignant cells.

## Data Availability

Data are contained within the article.
